# Crossing borders: Fostering early career international networks, critical for medical training and scientific research

**DOI:** 10.1016/j.amsu.2020.05.008

**Published:** 2020-05-18

**Authors:** Arash Ghaffari-Rafi, Jose Rojas-Leon, Sabahat Iqbal

**Affiliations:** a University of Hawai′i at Mānoa John A. Burns School of Medicine, Honolulu, HI, USA; b Universidad Internacional del Ecuador Escuela de Medicina, Quito, Ecuador; c University College London NHS National Hospital for Neurology and Neurosurgery, London, UK

Physician trainees generally study for years in a single country, yet medicine is an international endeavor.

Brought together for one year in an intercalated research MSc, we engaged in our first meaningful international networking experience, benefiting our career development and cultural maturity.

Today research is driven by international collaboration, with papers coauthored internationally having greater citation impact[[1], [2]]. Successful long-term collaborations generally establish after authors have previously worked together in the same physical facility on multiple occasions, allowing for significant face-to-face communication and behavioral interactions that are not possible digitally[3]. Our personal experiences in working together over one year has enabled us to create meaningful interpersonal relationships and hence, lead to successful international collaborations—for despite proceeding with our medical training, we continue to work together on clinical projects.

Studying amongst and as foreigners, we experienced cultural enrichment. We exposed each other to new ideas and perspectives, including learning about healthcare systems and medical difficulties faced in our respective countries—gaining unique insight applicable to our individual clinical practices. Submerged in interactions amongst diverse backgrounds increased our maturity and awareness of personal biases. Beyond the personal growth, in better empathizing with the clinical challenges faced outside our respective nations, we also developed a greater sense of inclusion as part of a global medical enterprise.

The opportunity for travel-exchange programs during or just prior to residency, where trainees from across the globe can converge at a point in their careers where similar interests are more likely to exist is critical for the development of meaningful networks. In turn, this will lead to improved clinician quality and therefore, better patient care. Our early career opportunity to intercalate an international research MSc into our medical education was fundamentally impactful on our clinical training—allowing for the establishment of our first meaningful international network.

## Funding

None.

## Disclosures

None reported.

## Contribution statement

The authors have equally contributed to the development of this article.

## Provenance and peer review

Not commissioned, Editor reviewed.

## Declaration of competing interest

We have read and understood the policy on declaration of interests and declare none.
